# Construction Notes

**Published:** 1895-11-09

**Authors:** 


### CONSTRUCTION NOTES.

### NEW ROYAL HOSPITAL FOR SICK CHILDREN, EDINBURGH.

The handsome new hospital which has been built at Meadowside, Edinburgh, for the treatment of sick children, has been now formally opened by H.R.H. Princess Beatrice. The block plan of the main hospital building is in the form of the letter E, the administrative block being in the centre of the building, and the wards in the two arms cut off from the centre by disconnecting cross ventilating corridors. It is set with its open side towards the south. This allows the sunshine to penetrate every part of the quadrangle. The administrative block is five storeys in height, the two upper storeys being in the roof, and the ward wings are three storeys in height. The principal wards are upon the ground and first floors, those upon the third floor being spare, observation, and isolation wards. The kitchen, with its accompanying larders, milk-house, and stores, is placed upon the fourth floor, and the dormitories and bed-room accommodation for the domestic staff is upon the fifth floor or attic. The main entrance is from the south, in the centre of the administrative block, by a large and well furnished hall, across which runs a 10 feet wide corridor extending right and left nearly the whole length of the building to the ward doors. Immediately to the right and left are the suite of rooms devoted to the matron and the resident doctors, while opposite the entrance hall, in the centre of the administrative block to the north, is the surgical theatre, with a gallery to accommodate 84 students. To the right of this is the principal staircase, to the left the service stairs, while beyond these, occupying the north-east and south-west corners of the administrative block, are rooms for the visiting physicians, one for each ward. A novel and attractive feature are the play alcoves for the convalescent children of each ward, formed in bays to the south, with large windows for the admission of sunshine. Each of the four principal wards is 84 feet long. They are arranged for 24 cots to each ward, and allow 1,250 cubic of air space to each child. The total accommodation is 118 beds. The angles of all walls and ceilings are rounded, and every precaution taken to avoid corners or ledges. In communication with each ward is a kitchen or pantry; bath-rooms, lavatories, slop and [sick rooms, &c., are also attached. The sanitary arrangements throughout the buildings, and especially those in connection with the wards, have received the greatest care and attention. The general arrangement for the ground floor is repeated upon the first floor, where the medical lecture theatre takes the place of the surgical one on the floor below, and the south range of rooms in the administrative block are devoted to the board-room, museum, ophthalmic-room, dispensary, assistant matron's room, staff dining-room, and nurses' sitting-room. On the second floor the wards are subdivided into observation, isolation, and spare wards, each with kitchen and lavatory accommodation. The front of the administrative block is devoted to a suite of bed-rooms and sitting-room for the sisters or staff nurses. The whole of the nursing staff are provided with separate bed-rooms. The building throughout is fitted with electric light. In addition to open fire-places in the wards and rooms a hot-water heating system is fitted throughout the building. The nurses' annexe and the pathological blocks are in close proximity to the main building, to which they are attached by covered corridors. The out-patients' department, laundry, and electric lighting buildings are detached from the hospital proper. The entire building has been erected from the designs and under the personal superintendence of the architect, Mr. G. Washington Browne, A.R.S.A. The cost of construction is about £40,000. The contractor for mason work was Mr. Hugh Mackintosh.

